# 1-Acetyl-4-(phenyl­sulfan­yl)imidazolidin-2-one

**DOI:** 10.1107/S1600536812007908

**Published:** 2012-02-29

**Authors:** Alaa A.-M. Abdel-Aziz, Adel S. El-Azab, Amer M. Alanazi, Seik Weng Ng, Edward R. T. Tiekink

**Affiliations:** a Department of Pharmaceutical Chemistry, College of Pharmacy, King Saud University, Riyadh 11451, Saudi Arabia; b Department of Medicinal Chemistry, Faculty of Pharmacy, University of Mansoura, Mansoura 35516, Egypt; c Department of Organic Chemistry, Faculty of Pharmacy, Al-Azhar University, Cairo 11884, Egypt; d Department of Chemistry, University of Malaya, 50603 Kuala Lumpur, Malaysia; e Chemistry Department, Faculty of Science, King Abdulaziz University, PO Box 80203 Jeddah, Saudi Arabia

## Abstract

The five-membered ring in the title imidazolidinone derivative, C_11_H_12_N_2_O_2_S, adopts an envelope conformation with the S-bound C atom being the flap atom. Overall, the mol­ecule has a U-shaped conformation as both rings are folded towards each other [dihedral angle = 31.66 (6)°]. An eight-membered amide {⋯HNCO}_2_ synthon leads to hydrogen-bonded dimeric aggregates in the crystal: these are additionally linked by C—H⋯π inter­actions.

## Related literature

For the anti­tumour potential of imidazolidinones, see: Abdel-Aziz *et al.* (2012 ▶). For ring conformational analysis, see: Cremer & Pople (1975 ▶).
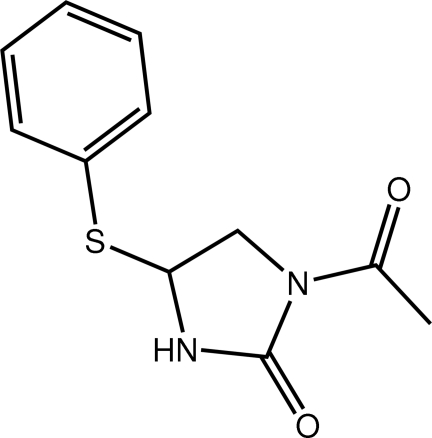


## Experimental

### 

#### Crystal data

C_11_H_12_N_2_O_2_S
*M* *_r_* = 236.29
Monoclinic, 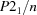

*a* = 7.0473 (1) Å
*b* = 14.3274 (3) Å
*c* = 10.7796 (2) Å
β = 96.921 (2)°
*V* = 1080.48 (3) Å^3^
*Z* = 4
Cu *K*α radiation
μ = 2.56 mm^−1^
*T* = 100 K
0.35 × 0.30 × 0.25 mm

#### Data collection

Agilent SuperNova Dual diffractometer with Atlas detector
Absorption correction: multi-scan (*CrysAlis PRO*; Agilent, 2011 ▶) *T*_min_ = 0.754, *T*_max_ = 1.000
8437 measured reflections
2186 independent reflections
2135 reflections with *I* > 2σ(*I*)
*R*_int_ = 0.015

#### Refinement

*R*[*F* ^2^ > 2σ(*F* ^2^)] = 0.028
*wR*(*F* ^2^) = 0.073
*S* = 1.01
2186 reflections
150 parameters
H atoms treated by a mixture of independent and constrained refinement
Δρ_max_ = 0.25 e Å^−3^
Δρ_min_ = −0.27 e Å^−3^

### 

Data collection: *CrysAlis PRO* (Agilent, 2011 ▶); cell refinement: *CrysAlis PRO*; data reduction: *CrysAlis PRO*; program(s) used to solve structure: *SHELXS97* (Sheldrick, 2008 ▶); program(s) used to refine structure: *SHELXL97* (Sheldrick, 2008 ▶); molecular graphics: *ORTEP-3* (Farrugia, 1997 ▶) and *DIAMOND* (Brandenburg, 2006 ▶); software used to prepare material for publication: *publCIF* (Westrip, 2010 ▶).

## Supplementary Material

Crystal structure: contains datablock(s) global, I. DOI: 10.1107/S1600536812007908/bt5824sup1.cif

Structure factors: contains datablock(s) I. DOI: 10.1107/S1600536812007908/bt5824Isup2.hkl

Supplementary material file. DOI: 10.1107/S1600536812007908/bt5824Isup3.cml

Additional supplementary materials:  crystallographic information; 3D view; checkCIF report

## Figures and Tables

**Table 1:** Hydrogen-bond geometry (Å, °) *Cg*1 is the centroid of the C6–C11 ring.

*D*—H⋯*A*	*D*—H	H⋯*A*	*D*⋯*A*	*D*—H⋯*A*
N2—H1⋯O1^i^	0.876 (19)	2.032 (19)	2.8989 (13)	169.8 (17)
C1—H1*A*⋯*Cg*1^ii^	0.98	2.72	3.6360 (13)	155

Symmetry codes: (i) 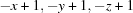; (ii) 
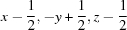
.

**Fig. 1.**
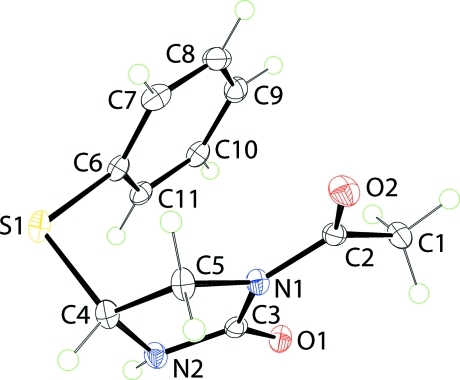
The molecular structure of (I) showing the atom-labelling scheme and displacement ellipsoids at the 50% probability level.

**Fig. 2.**
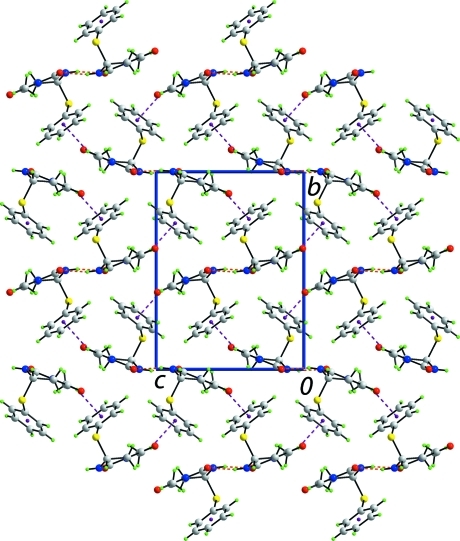
A view in projection down the a axis of the unit-cell contents for (I). The N—H···O and C—H···π interactions are shown as orange and purple dashed lines, respectively.

## supplementary crystallographic information

### Comment

A recent study described the anti-tumor potential of imidazolidinones (Abdel-Aziz *et al.*, 2012). In continuation of these studies, herein, the crystal structure determination of an imidazolidinone derivative, 1-acetyl-4-(phenylthio)imidazolidin-2-one (I) is described.

The five-membered ring in (I), Fig. 1, adopts an envelope conformation with the C4 atom being the flap atom. The puckering parameters (Cremer & Pople, 1975) are Q = 0.2364 (12) Å and φ_2_ = 113.9 (3)°. The molecule has a U-shaped conformation whereby the five- and six-membered rings lie to the same side of the molecule and form a dihedral angle of 31.66 (6)°.

In the crystal packing, centrosymmetrically related molecules associate *via* N—H···O hydrogen bonds leading to the familiar eight-membered amide {···HNCO}_2_ synthon, Table 1. The dimers are connected into the three-dimensional architecture by C—H···π interactions, Fig. 2 and Table 1.

### Experimental

At room temperature, trifluoroacetic acid (0.3 equiv.) was added drop wise to a stirred solution of 1-acetyl-4-methoxyimidazolidin-2-one (1 equiv.) and thiophenol (1 equiv.) in dry CH_3_CN (0.01 mol/l) over a period of 15 min. After being stirred for 2 h at room temperature, the mixture was quenched by adding ammonium chloride solution (5 ml). The product was extracted with ethylacetate, washed with brine and dried over anhydrous sodium sulfate. The product obtained after evaporation of solvent was purified by column chromatography using a mixture of hexane and CHCl_3_ (1:1 *v*/*v*) as eluent. Crystals were obtained by slow evaporation of the eluent solution. Yield, 96%. m.p. 383–384 K. IR (KBr, cm^-1^): ν 3320 (N—H), 1760, 1710 (C═ O). ^1^H NMR (CDCl_3_): δ 2.20 (s, 3H), 3.98 (m, 1H), 4.06 (m, 1H), 4.901 (m, 1H), 6.42 (s, 1H), 7.28 (d, 3H, J = 7.0 Hz), 7.45–7.46 (d, 2H, J = 5.5 Hz). ^13^C NMR (CDCl_3_): δ 23.21, 48.95, 56.17, 127.51, 129.07, 129.36, 129.46, 135.22, 155.12, 170.11.

### Refinement

Carbon-bound H atoms were placed in calculated positions [C—H = 0.95 to 1.00 Å, *U*_iso_(H) = 1.2–1.5*U*_eq_(C)] and were included in the refinement in the riding model approximation. The H atom bonded to N was freely refined.

### Crystal data

**Table:**

C_11_H_12_N_2_O_2_S	*F*(000) = 496
*M**_r_* = 236.29	*D*_x_ = 1.453 Mg m^−^^3^
Monoclinic, *P*2_1_/*n*	Cu *K*α radiation, λ = 1.5418 Å
Hall symbol: -P 2yn	Cell parameters from 6092 reflections
*a* = 7.0473 (1) Å	θ = 3.1–76.4°
*b* = 14.3274 (3) Å	µ = 2.56 mm^−^^1^
*c* = 10.7796 (2) Å	*T* = 100 K
β = 96.921 (2)°	Prism, colourless
*V* = 1080.48 (3) Å^3^	0.35 × 0.30 × 0.25 mm
*Z* = 4	

### Data collection

**Table:**

Agilent SuperNova Dual diffractometer with Atlas detector	2186 independent reflections
Radiation source: SuperNova (Cu) X-ray Source	2135 reflections with *I* > 2σ(*I*)
Mirror monochromator	*R*_int_ = 0.015
Detector resolution: 10.4041 pixels mm^-1^	θ_max_ = 76.6°, θ_min_ = 5.2°
ω scans	*h* = −8→8
Absorption correction: multi-scan (*CrysAlis PRO*; Agilent, 2011)	*k* = −17→15
*T*_min_ = 0.754, *T*_max_ = 1.000	*l* = −10→13
8437 measured reflections	

### Refinement

**Table:**

Refinement on *F*^2^	Primary atom site location: structure-invariant direct methods
Least-squares matrix: full	Secondary atom site location: difference Fourier map
*R*[*F*^2^ > 2σ(*F*^2^)] = 0.028	Hydrogen site location: inferred from neighbouring sites
*wR*(*F*^2^) = 0.073	H atoms treated by a mixture of independent and constrained refinement
*S* = 1.01	*w* = 1/[σ^2^(*F*_o_^2^) + (0.0413*P*)^2^ + 0.5578*P*] where *P* = (*F*_o_^2^ + 2*F*_c_^2^)/3
2186 reflections	(Δ/σ)_max_ = 0.001
150 parameters	Δρ_max_ = 0.25 e Å^−^^3^
0 restraints	Δρ_min_ = −0.27 e Å^−^^3^

### Special details

**Table:**

Geometry. All e.s.d.'s (except the e.s.d. in the dihedral angle between two l.s. planes) are estimated using the full covariance matrix. The cell e.s.d.'s are taken into account individually in the estimation of e.s.d.'s in distances, angles and torsion angles; correlations between e.s.d.'s in cell parameters are only used when they are defined by crystal symmetry. An approximate (isotropic) treatment of cell e.s.d.'s is used for estimating e.s.d.'s involving l.s. planes.
Refinement. Refinement of *F*^2^ against ALL reflections. The weighted *R*-factor *wR* and goodness of fit *S* are based on *F*^2^, conventional *R*-factors *R* are based on *F*, with *F* set to zero for negative *F*^2^. The threshold expression of *F*^2^ > σ(*F*^2^) is used only for calculating *R*-factors(gt) *etc*. and is not relevant to the choice of reflections for refinement. *R*-factors based on *F*^2^ are statistically about twice as large as those based on *F*, and *R*-factors based on ALL data will be even larger.

### Fractional atomic coordinates and isotropic or equivalent isotropic displacement parameters (Å^2^)

**Table:**

	*x*	*y*	*z*	*U*_iso_*/*U*_eq_	
S1	0.90697 (4)	0.65626 (2)	0.40810 (3)	0.01816 (10)	
O1	0.34661 (12)	0.49945 (6)	0.34757 (8)	0.01716 (19)	
O2	0.48246 (13)	0.61074 (6)	0.00853 (8)	0.0195 (2)	
N1	0.54386 (14)	0.54954 (7)	0.20071 (9)	0.0138 (2)	
N2	0.67635 (15)	0.49985 (7)	0.38573 (9)	0.0161 (2)	
C1	0.20799 (17)	0.57746 (9)	0.11228 (11)	0.0174 (3)	
H1A	0.1359	0.6028	0.0363	0.026*	
H1B	0.1814	0.6146	0.1846	0.026*	
H1C	0.1696	0.5126	0.1237	0.026*	
C2	0.41743 (17)	0.58111 (8)	0.10054 (10)	0.0142 (2)	
C3	0.50542 (17)	0.51505 (8)	0.31596 (10)	0.0138 (2)	
C4	0.83250 (17)	0.54464 (9)	0.33269 (11)	0.0163 (2)	
H4	0.9445	0.5013	0.3399	0.020*	
C5	0.75037 (17)	0.55287 (9)	0.19467 (11)	0.0169 (2)	
H5A	0.7883	0.6124	0.1582	0.020*	
H5B	0.7925	0.5003	0.1450	0.020*	
C6	0.68485 (17)	0.71643 (8)	0.38907 (11)	0.0154 (2)	
C7	0.63946 (19)	0.77629 (9)	0.28787 (11)	0.0192 (3)	
H7	0.7304	0.7878	0.2314	0.023*	
C8	0.4618 (2)	0.81908 (9)	0.26958 (12)	0.0209 (3)	
H8	0.4305	0.8590	0.1997	0.025*	
C9	0.32924 (19)	0.80386 (9)	0.35317 (12)	0.0201 (3)	
H9	0.2073	0.8329	0.3402	0.024*	
C10	0.37606 (18)	0.74601 (9)	0.45571 (11)	0.0180 (3)	
H10	0.2862	0.7362	0.5134	0.022*	
C11	0.55324 (17)	0.70240 (8)	0.47448 (11)	0.0158 (2)	
H11	0.5848	0.6632	0.5450	0.019*	
H1	0.675 (3)	0.4930 (13)	0.4664 (18)	0.032 (5)*	

### Atomic displacement parameters (Å^2^)

**Table:**

	*U*^11^	*U*^22^	*U*^33^	*U*^12^	*U*^13^	*U*^23^
S1	0.01240 (17)	0.02410 (18)	0.01774 (16)	−0.00284 (10)	0.00085 (11)	−0.00434 (11)
O1	0.0171 (5)	0.0213 (4)	0.0130 (4)	−0.0053 (3)	0.0016 (3)	0.0022 (3)
O2	0.0216 (5)	0.0235 (5)	0.0139 (4)	−0.0003 (4)	0.0039 (3)	0.0045 (3)
N1	0.0132 (5)	0.0171 (5)	0.0112 (4)	−0.0003 (4)	0.0018 (4)	0.0004 (4)
N2	0.0167 (5)	0.0189 (5)	0.0121 (5)	0.0000 (4)	−0.0005 (4)	0.0016 (4)
C1	0.0154 (6)	0.0203 (6)	0.0163 (5)	0.0016 (5)	0.0003 (4)	0.0031 (4)
C2	0.0178 (6)	0.0123 (5)	0.0122 (5)	0.0004 (4)	0.0003 (4)	−0.0004 (4)
C3	0.0181 (6)	0.0112 (5)	0.0117 (5)	−0.0011 (4)	0.0004 (4)	−0.0010 (4)
C4	0.0141 (6)	0.0199 (6)	0.0145 (5)	0.0016 (4)	0.0003 (4)	−0.0017 (4)
C5	0.0132 (6)	0.0239 (6)	0.0137 (5)	0.0005 (5)	0.0013 (4)	−0.0024 (5)
C6	0.0152 (6)	0.0160 (6)	0.0148 (5)	−0.0041 (4)	0.0018 (4)	−0.0045 (4)
C7	0.0252 (7)	0.0177 (6)	0.0158 (6)	−0.0051 (5)	0.0065 (5)	−0.0022 (4)
C8	0.0300 (7)	0.0150 (6)	0.0170 (6)	−0.0016 (5)	−0.0001 (5)	0.0010 (5)
C9	0.0189 (6)	0.0175 (6)	0.0231 (6)	0.0004 (5)	−0.0006 (5)	−0.0038 (5)
C10	0.0179 (6)	0.0189 (6)	0.0178 (6)	−0.0042 (5)	0.0046 (5)	−0.0045 (5)
C11	0.0187 (6)	0.0159 (6)	0.0128 (5)	−0.0046 (5)	0.0015 (4)	−0.0015 (4)

### Geometric parameters (Å, º)

**Table:**

S1—C6	1.7771 (13)	C4—H4	1.0000
S1—C4	1.8413 (13)	C5—H5A	0.9900
O1—C3	1.2290 (15)	C5—H5B	0.9900
O2—C2	1.2181 (14)	C6—C7	1.3943 (18)
N1—C3	1.3936 (14)	C6—C11	1.3978 (16)
N1—C2	1.3902 (15)	C7—C8	1.3864 (19)
N1—C5	1.4654 (15)	C7—H7	0.9500
N2—C3	1.3586 (16)	C8—C9	1.3913 (18)
N2—C4	1.4496 (15)	C8—H8	0.9500
N2—H1	0.876 (19)	C9—C10	1.3891 (18)
C1—C2	1.4975 (16)	C9—H9	0.9500
C1—H1A	0.9800	C10—C11	1.3891 (18)
C1—H1B	0.9800	C10—H10	0.9500
C1—H1C	0.9800	C11—H11	0.9500
C4—C5	1.5346 (16)		
			
C6—S1—C4	99.80 (6)	N1—C5—C4	102.41 (9)
C3—N1—C2	129.24 (10)	N1—C5—H5A	111.3
C3—N1—C5	110.61 (10)	C4—C5—H5A	111.3
C2—N1—C5	120.12 (10)	N1—C5—H5B	111.3
C3—N2—C4	112.04 (10)	C4—C5—H5B	111.3
C3—N2—H1	116.8 (12)	H5A—C5—H5B	109.2
C4—N2—H1	122.8 (12)	C7—C6—C11	119.73 (12)
C2—C1—H1A	109.5	C7—C6—S1	120.30 (9)
C2—C1—H1B	109.5	C11—C6—S1	119.96 (10)
H1A—C1—H1B	109.5	C8—C7—C6	120.09 (11)
C2—C1—H1C	109.5	C8—C7—H7	120.0
H1A—C1—H1C	109.5	C6—C7—H7	120.0
H1B—C1—H1C	109.5	C7—C8—C9	120.26 (12)
O2—C2—N1	118.50 (11)	C7—C8—H8	119.9
O2—C2—C1	123.53 (11)	C9—C8—H8	119.9
N1—C2—C1	117.97 (10)	C8—C9—C10	119.68 (12)
O1—C3—N2	126.42 (10)	C8—C9—H9	120.2
O1—C3—N1	126.36 (11)	C10—C9—H9	120.2
N2—C3—N1	107.20 (10)	C11—C10—C9	120.49 (11)
N2—C4—C5	101.59 (9)	C11—C10—H10	119.8
N2—C4—S1	113.57 (8)	C9—C10—H10	119.8
C5—C4—S1	114.55 (9)	C10—C11—C6	119.70 (11)
N2—C4—H4	108.9	C10—C11—H11	120.1
C5—C4—H4	108.9	C6—C11—H11	120.1
S1—C4—H4	108.9		
			
C3—N1—C2—O2	178.27 (11)	C3—N1—C5—C4	−16.59 (12)
C5—N1—C2—O2	0.41 (17)	C2—N1—C5—C4	161.64 (10)
C3—N1—C2—C1	−1.26 (18)	N2—C4—C5—N1	23.05 (12)
C5—N1—C2—C1	−179.12 (10)	S1—C4—C5—N1	−99.80 (10)
C4—N2—C3—O1	−167.06 (11)	C4—S1—C6—C7	−95.17 (10)
C4—N2—C3—N1	14.33 (13)	C4—S1—C6—C11	83.57 (10)
C2—N1—C3—O1	5.9 (2)	C11—C6—C7—C8	−2.31 (18)
C5—N1—C3—O1	−176.12 (11)	S1—C6—C7—C8	176.44 (9)
C2—N1—C3—N2	−175.53 (11)	C6—C7—C8—C9	1.12 (19)
C5—N1—C3—N2	2.49 (13)	C7—C8—C9—C10	0.44 (19)
C3—N2—C4—C5	−23.91 (13)	C8—C9—C10—C11	−0.80 (19)
C3—N2—C4—S1	99.60 (10)	C9—C10—C11—C6	−0.39 (18)
C6—S1—C4—N2	−55.80 (9)	C7—C6—C11—C10	1.94 (18)
C6—S1—C4—C5	60.32 (9)	S1—C6—C11—C10	−176.81 (9)

### Hydrogen-bond geometry (Å, º)

Cg1 is the centroid of the C6–C11 ring.

**Table:**

*D*—H···*A*	*D*—H	H···*A*	*D*···*A*	*D*—H···*A*
N2—H1···O1^i^	0.876 (19)	2.032 (19)	2.8989 (13)	169.8 (17)
C1—H1*A*···*Cg*1^ii^	0.98	2.72	3.6360 (13)	155

Symmetry codes: (i) −*x*+1, −*y*+1, −*z*+1; (ii) *x*−1/2, −*y*+1/2, *z*−1/2.
